# Structure and Photoluminescence Properties of Dy^3+^ Doped Phosphor with Whitlockite Structure

**DOI:** 10.3390/ma15062177

**Published:** 2022-03-15

**Authors:** Wanjuan Tang, Qingfeng Guo, Ke Su, Haikun Liu, Yuanyuan Zhang, Lefu Mei, Libing Liao

**Affiliations:** 1 School of Gemmology, China University of Geosciences, Beijing 100083, China; 1009191121@cugb.edu.cn; 2 Beijing Key Laboratory of Materials Utilization of Nonmetallic Minerals and Solid Wastes, National Laboratory of Mineral Materials, School of Materials Sciences and Technology, China University of Geosciences, Beijing 100083, China; kesu@cugb.edu.cn; 3 School of Chemical Engineering and Energy Technology, Dongguan University of Technology, Dongguan 523808, China; hkliu7274@126.com (H.L.); zhangyy4192@126.com (Y.Z.); 4 Science and Technology Innovation Institute, Faculty of Science, Dongguan University of Technology, Dongguan 523808, China

**Keywords:** phosphor, whitlockite, Dy^3+^

## Abstract

Whitlockite has the advantages of a low sintering temperature, high stability, and a low fabrication cost, and it is widely used as the host for luminescent material. In this study, Ca_1.8_Li_0.6_La_0.6−x_(PO_4_)_2_:xDy^3+^ phosphor was prepared by the high-temperature solid-state method, and its structure, composition, and luminescence properties were systematically studied. The results showed that a new whitlockite type matrix was prepared by replacing Ca^2+^ in whitlockite with monovalent and trivalent cations. The prepared phosphors belonged to a hexagonal crystal system with a particle size in the range of 5–20 μm. Under the excitation of 350 nm UV light, the samples emitted white light, and there were mainly two stronger emission peaks at 481 nm in the blue band and 573 nm in the yellow band, which correspond to the electron transitions at ^4^F_9/2_→^6^H_15/2_ and ^4^F_9/2_→^6^H_13/2_ of Dy^3+^, respectively. The optimal doping concentration of Dy^3+^ in Ca_1.8_Li_0.6_La_0.6_(PO_4_)_2_ matrix was 0.03 (mol%). The main mechanism of concentration quenching in the sample was dipole–dipole energy transfer. When the temperature was 130 °C, the luminescence intensity of the samples was 78.7% of that at 30 °C, and their thermal quenching activation energy was 0.25 eV. The CIE coordinates of the sample at 30 °C were (0.2750, 0.3006), and their luminescent colors do not change with temperature. All the results indicate that Ca_1.8_Li_0.6_La_0.6__−x_(PO_4_)_2_:xDy^3+^ phosphor is a luminescent material with good luminescence performance and thermal stability, which shows a promising application in the field of LED display.

## 1. Introduction

Rare earth materials are essential materials for many highly sophisticated industries today, and they have their own unique advantages in many aspects. There has been extensive research on rare earths in permanent magnetic materials [1], luminescent materials [2,3], catalytic materials [4,5,6], and hydrogen storage materials [7]. With the development of science and technology, rare-earth luminescent materials often appear in the research of luminescent materials for third-generation lamps. Rare-earth luminescent materials have a high luminescence efficiency, a strong emission ability in the visible region, resistance to ultraviolet radiation, good thermal stability, and can adapt to the requirements of high-load fluorescent lamps. Therefore, rare-earth luminescent materials are widely used in fluorescent lamps, white diode fluorescents, display imaging, X-ray enhancement screens, and other fields.

Host and activator make up the majority of rare-earth luminous materials. As a host, the most basic needs are stability and ease of preparation. Whitlockite (β-Ca_3_(PO_4_)_2_) has the advantages of a low sintering temperature, a low manufacturing cost, and strong physical and chemical stability [8,9]. On the other hand, there are five non-equivalent cation lattice sites that exist for rare-earth ions in the whitlockite structure. Therefore, whitlockite is an outstanding host. Isomorphism can open up new possibilities for luminous materials and is crucial for the discovery of new luminescent materials. Lithium is an alkaline earth metal with a solubilization function that can boost the luminescence of rare-earth ions significantly. La^3+^ is commonly employed in the fabrication of luminous matrix materials doped with rare-earth ions because it has a stable structure with nearly negligible light absorption and emission. As a result, Li^+^ and La^3+^ have frequently been utilized to micro-modify the structure of the luminous matrix, such as Li_7_La_3_Zr_2_O_12_:xTb [10], Li_7_La_3__−x_Zr_2_O_12_:xEu^3+^ [11], Li_5_La_3_Nb_2_O_12_ [12], and La_2_LiMO_6_:Eu^3+^ [13]. Scholars at home and abroad have been paying close attention to the design and development of luminous materials using whitlockite materials as matrices in recent years [2,14,15]. The introduction of Li^+^ and La^3+^ in whitlockite structure may bring some new opportunities to generate novel excellent whitlockite host for luminescent materials. In conclusion, luminous materials with the matrix Ca_1.8_Li_0.6_La_0.6_(PO_4_)_2_ were created in this article.

Activators in luminous materials activate the host by forming luminescent centers. To generate prepared luminous materials with good luminescent characteristics, rare-earth ions are frequently doped in the host as activators. Dy^3+^ is a rare-earth ion that is commonly employed to make luminous materials [16,17,18,19]. ^4^F_9/2_→^6^H_13/2_ transitions are found in the yellow zone of Dy^3+^ ions, while ^4^F_9/2_→^6^H_15/2_ transitions are found in the blue region. When the Dy^3+^ ion is at the center of symmetry, multilevel interactions are the main cause of luminescence, and the luminescent material emits standard white light when the transitions intensity is comparable in both places [20,21].

Currently, most commercial white LEDs are manufactured by using blue diode chips to excite the yellow phosphor Y_3_Al_5_O_12_:Ce^3+^ [22]. Due to the absence of the red spectral component, white light from this combination exhibits poor color rendering index and high color temperature, and the white light emitted is unstable. Single-component white phosphor has better color stability, it produces less energy loss during energy transfer, and the pure white color is also more favorable for phosphor color improvement. Therefore, the development of new single-component white phosphor has obvious practical application value.

In this study, single-component phosphors, Ca_1.8_Li_0.6_La_0.6__−x_(PO_4_)_2_:xDy^3+^, were prepared by the high-temperature solid state method. Their composition, structure, and luminescent properties were systematically investigated using a combination of X-ray diffractometer, scanning electron microscope, transmission electron microscope, and fluorescence spectrometer testing techniques. The findings reveal that the luminous material is a white phosphor with good luminescence performance and thermal stability, indicating that it has significant research potential.

## 2. Materials and Methods

Ca_1.8_Li_0.6_La_0.6__−x_(PO_4_)_2_:xDy^3+^ phosphors (x = 0, 0.01, 0.03, 0.06, 0.09, 0.12, 0.15, 0.18, and 0.21) were synthesized using a high-temperature solid-phase technique in this study. The raw ingredients, Li_2_CO_3_ (Macklin, Shanghai, China, 99.9%), La_2_O_3_ (Aladdin, Shanghai, China, 99.9%), NH_4_H_2_PO_4_ (Macklin, Shanghai, China, 99.9%), CaCO_3_ (Aladdin, Shanghai, China, 99.9%), and Dy_2_O_3_ (Aladdin, Shanghai, China, 99.999%) were correctly weighed according to the structural stoichiometric ratios. The quality of raw materials is shown in Table 1. The weighed samples were pulverized for around 15 min in an agate mortar before being poured into an alumina crucible after they were entirely ground and combined. The samples were placed in a muffle furnace (Kejing Material Technology Co., Ltd., Hefei, China), ramped up to 650 °C at a rate of 5 °C/min, and placed at 650 °C for 1 h in an air atmosphere. After the samples were cooled to room temperature, they were removed and ground to a powder. Then, it was placed in a tube furnace (GXL-1700X, Kejing Material Technology Co., Ltd., Hefei, China) and heated up to 1250 °C at 10 °C/min and left at 1250 °C for 4 h in an air atmosphere. After the temperature of the tube furnace was reduced to room temperature, the sample was transferred to a mortar for fine grinding to obtain the test sample.

XRD patterns were obtained on a BRUKER D8 ADVANCE powder diffractometer (Bruker, Karlsruhe, Baden-Württemberg, Germany) with Cu Kα radiation (λ = 0.15418 nm), continuous scanning mode, 40 mA and 40 kV, and a 2θ range of 10–70°. EDAX TEAM spectrometers (EDAX, Pleasanton, California, USA) of the Octane Pro, Plus, Super, and Ultra versions were used to measure the phosphors’ SEM spectra. A transmission electron microscope model JEM-2100F (JEOL, Tokyo, Japan) was used to measure the TEM of the samples. A fluorescence spectrometer, FL4600 (HITACHI, Tokyo, Japan), with a 150 V xenon excitation source and a photomultiplier voltage of 400 V, was used to measure the phosphors’ excitation (PLE) and emission (PL) spectra. Variable temperature emission spectra of the samples were obtained using a spectrometer with a digital temperature controller and a 600 V photomultiplier voltage.

## 3. Results

### 3.1. Crystal Structure

**Figure 1:**
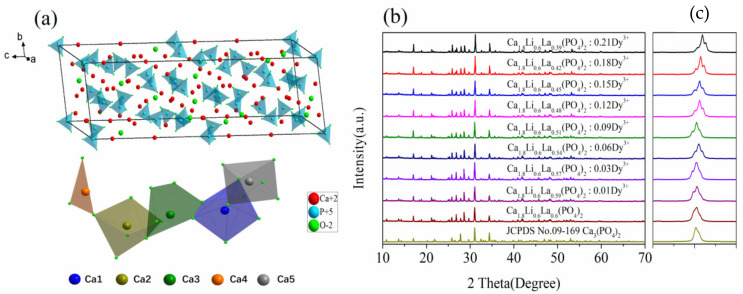
(**a**) Cell structure diagram of β-Ca_3_(PO_4_)_2_, (**b**,**c**) XRD patterns of Ca_1.8_Li_0.6_La_0.6__−x_(PO_4_)_2_:xDy^3+^ phosphor and JCPDS No.09-169.

Ca_3_(PO_4_)_2_ (JCPDS No. 09-0169) is a hexagonal crystal system that belongs to the R3c (167) space group, and its crystal structure is depicted in Figure 1a, drawn by Diamond software (University of Bonn, Bonn, North Rhine-Westphalia, Germany). As shown in Figure 1a, whitlockite has five cationic sites Ca1–Ca5, with Ca1, Ca2, and Ca3 occupying general positions, while Ca4 and Ca5 occupy positions 6a and 6b on the triple rotation axis, respectively. By substituting Ca^2+^, different whitlockite compounds can be made.

X-ray diffractograms (XRD) of the sample Ca_1.8_Li_0.6_La_0.6__−x_(PO_4_)_2_:xDy^3+^ (x = 0, 0.01, 0.03, 0.06, 0.09, 0.12, 0.15, 0.18, and 0.21) and the standard card JCPDS No. 09-169 are shown in Figure 1b. The diffraction peaks of the samples match well with that of the standard card for Ca_3_(PO_4_)_2_ (JCPDS No. 09-0169). The resulting sample is still pure phase despite replacing the portion of the Ca^2+^ by Li^+^ and La^3+^. The sample Ca_1.8_Li_0.6_La_0.6__−x_(PO_4_)_2_:xDy^3+^ has roughly the same structure as the Ca_3_(PO_4_)_2_ and belongs to the hexagonal crystal system with the R3c (167) space group. The local magnification of the sample from 30° to 32° is shown in Figure 1c, and it is clear that the strongest diffraction peaks shifted to a higher angle when the Dy^3+^ doping concentration increases, due to the fact that the smaller radius Dy^3+^ (CN = 6, R_Dy_^3+^ = 0.908 Å) occupies the crystal lattice of the larger radius La^3+^ (CN = 6, R_La_^3+^ = 1.061 Å).

To learn more about the structure of the samples, we analyzed the XRD results and obtained the lattice parameters, which we compared to the standard card JCPDS No. 09-169, and the results are displayed in Table 2. In the hexagonal crystal structure, a = b = 90°, and c = 120°. The c-axis of the sample gradually shortens with the increase in the Dy^3+^ doping concentration, and the cell volume steadily reduces, as shown in Table 2. This also means that Dy^3+^ was successfully absorbed into the crystal structure.

To further understand the crystalline nature of the samples, their average crystallite size was calculated using the Scherrer equation:

(1) $$D=\frac{k\lambda }{\beta \cos\theta }$$

where k is the Scherrer constant (k = 0.89), *λ* is the wavelength of radiation (*λ* = 0.15418), and *β* is the full width at half maximum parameter of the measured sample. We calculated the XRD data of Ca_1.8_Li_0.6_La_0.57_(PO_4_)_2_:0.03Dy^3+^ and obtained its average crystallite size as 63.92 nm.

**Figure 2:**
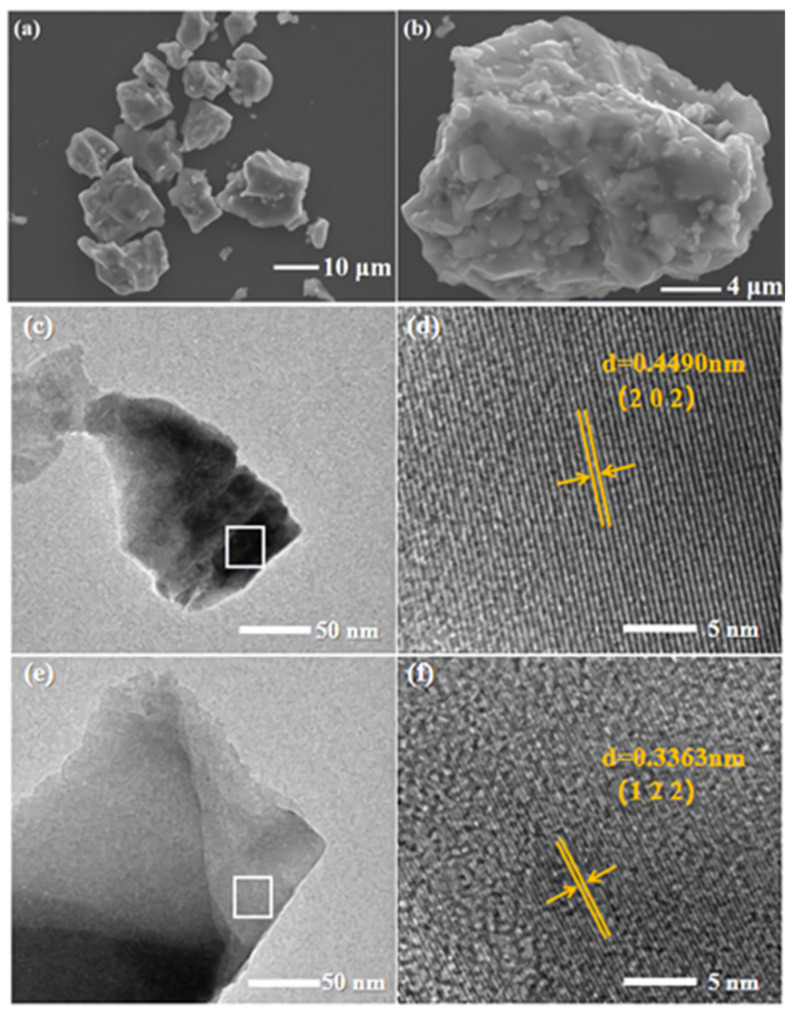
(**a**,**b**) SEM images of Ca_1.8_Li_0.6_La_0.6_(PO_4_)_2_ phosphor, (**c**–**f**) TEM and HRTEM image of Ca_1.8_Li_0.6_La_0.6_(PO_4_)_2_ phosphor and Ca_1.8_Li_0.6_La_0.57_(PO_4_)_2_:0.03Dy^3+^ phosphor.

Transmission electron micrographs (TEM) of Ca_1.8_Li_0.6_La_0.6_(PO_4_)_2_ and Ca_1.8_Li_0.6_La_0.57_(PO_4_)_2_:0.03Dy^3+^, as well as high-resolution transmission electron micrographs (HRTEM) of the designated regions, are shown in Figure 2c–f. According to HRTEM measurements, the crystalline surface spacing of the matrix was 0.4490 nm, which is consistent with the (202) crystalline surface of Ca_3_(PO_4_)_2_ (JCPDS No. 09-0169), and the crystalline surface spacing of the Dy^3+^-doped sample was 0.3363 nm, which is consistent with the (122) crystalline surface of Ca_3_(PO_4_)_2_, indicating that the samples are well crystallized. Scanning electron microscopy (SEM) images of Ca_1.8_Li_0.6_La_0.6_(PO_4_)_2_ are shown in Figure 2a,b. The prepared matrix samples were irregular, well crystallized, without agglomerates, and with good dispersion. The surface of the samples was not flat, and their particle size was not uniform, ranging from 5 to 20 microns, which met the requirements of practical application.

### 3.2. CIE

**Figure 3:**
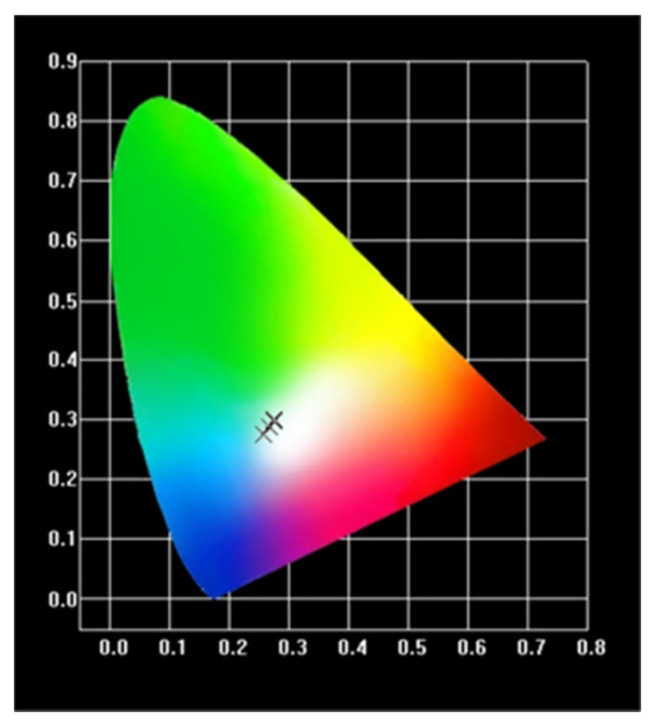
CIE coordinates diagram of Ca_1.8_Li_0.6_La_0.57_(PO_4_)_2_:0.03Dy^3+^. Phosphor at different temperatures.

Figure 3 and Table 3 show the CIE coordinates of Ca_1.8_Li_0.6_La_0.57_(PO_4_)_2_:0.03Dy^3+^ at various temperatures. The samples emit white light when the excitation wavelength is 350 nm, and their luminescence color does not change with temperature. Compared with the previous results of CaSr_2_(PO_4_)_2_:Dy^3+^,Li^+^ with CIE coordinates of (0.3450, 0.3787) [2], the present experimental sample emits a more standard white light.

### 3.3. Photoluminescence Characteristics

**Figure 4:**
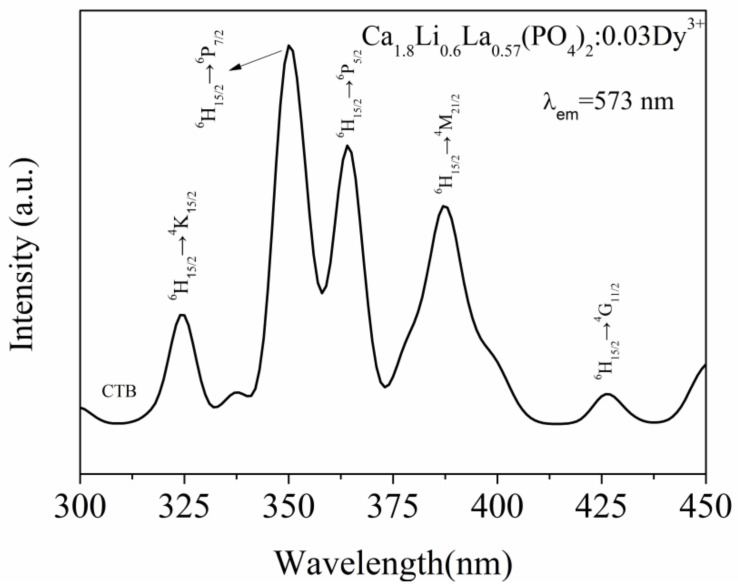
Photoluminescence excitation spectrum of Ca_1.8_Li_0.6_La_0.6__−x_(PO_4_)_2_:xDy^3+^ phosphor.

The photoluminescence excitation spectrum of Ca_1.8_Li_0.6_La_0.57_(PO_4_)_2_:0.03Dy^3+^ phosphor (λ_em_ = 573 nm) is shown in Figure 4. The sample’s largest excitation peak is at 350 nm, while the second strongest excitation peak is at 364 nm, which correspond to the electron transitions at ^6^H_15/2_→^6^P_7/2_ and ^6^H_15/2_→^6^P_5/2_ of Dy^3+^, respectively. Furthermore, Dy^3+^ has characteristic excitation peaks at 324 nm, 387 nm, and 426 nm, which correspond to the electron transitions at ^6^H_15/2_→^4^K_15/2_, ^6^H_15/2_→^4^M_21/2_, and ^6^H_15/2_→^4^G_11/2_ of Dy^3+^ [23], respectively, and the O^2−^→Dy^3+^ charge migration band exists in the region before 320 nm (CTB).

**Figure 5:**
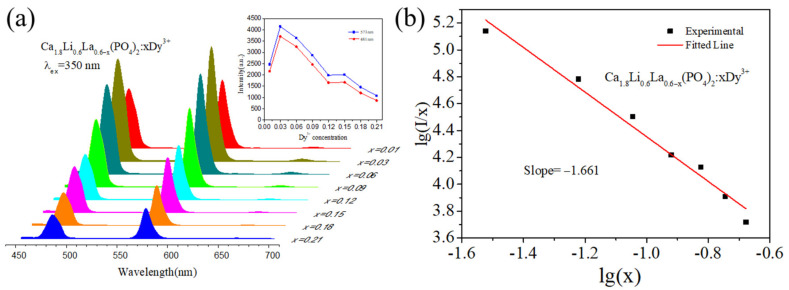
(**a**) Photoluminescence emission spectra of Ca_1.8_Li_0.6_La_0.6__−x_(PO_4_)_2_:xDy^3+^(x = 0.01, 0.03, 0.06, 0.09, 0.12, 0.15, 0.18, and 0.21) phosphor. (**b**) The relationship between. lg (I/x) and lg(x) of Ca_1.8_Li_0.6_La_0.6__−x_(PO_4_)_2_:xDy^3+^ phosphor.

The photoluminescence emission spectra of Ca_1.8_Li_0.6_La_0.6__−x_(PO_4_)_2_:xDy^3+^ (x = 0.01, 0.03, 0.06, 0.09, 0.12, 0.15, 0.18, and 0.21) phosphor samples at 350 nm are shown in Figure 5a. The figure shows that in the 450–700 nm band, there are mainly two stronger emission peaks at 481 nm in the blue band and 573 nm in the yellow band, which correspond severally to the electron transitions at ^4^F_9/2_→^6^H_15/2_ and ^4^F_9/2_→^6^H_13/2_ of Dy^3+^ [24]. A weaker emission peak at 662 nm in the red band corresponds to the electron transition at ^4^F_9/2_→^6^H_11/2_ of Dy^3+^. The connection between Dy^3+^ doping concentration and emission intensity at 481 nm and 573 nm is shown in the inset of Figure 5a.

Concentration quenching happened when the doping concentration of Dy^3+^ ions was 0.03. To investigate the mechanism of energy transfer between Dy^3+^ ions, we calculated the critical distance. The critical radius distance Rc can be calculated using the equation in Blasse theory [25] as follows:

(2) $$R_{c}\approx 2{\left[\frac{3V}{4\pi X_{C}N}\right]}^{\frac{1}{3}}$$

where *V* represents the volume per unit cell (*V* = 3509.69 Å), *N* represents the number of cations in a single cell (*N* = 21), and *Xc* represents the Dy^3+^ critical concentration (*X_C_* = 0.03). According to the calculations, *Rc* = 21.99 Å. The nonradiative energy transfer of ions, which is primarily caused by exchange interactions or multilayer interactions, is the principal source of concentration quenching. When the activator and sensitizer occupy nearby lattice sites and the overlap of their wave functions is adequate for electron exchange, i.e., when the critical distance between the activator and sensitizer is less than 5 Å, non-radiative energy transfer occurs. The values for *Rc* were substantially greater than 5 Å, indicating that multilevel interactions are used to transmit energy between Dy^3+^-Dy^3+^ ions in Ca_1.8_Li_0.6_La_0.6__−x_(PO_4_)_2_:xDy^3+^. The Dexter theoretical model states:

(3) $$\frac{I}{x}=K{\left[1+\beta {\left(x\right)}^{\theta /3}\right]}^{-1}$$

where *I* is the intensity of the emission, *x* is the concentration of Dy^3+^, and *K* and *β* are constants. The value of *θ* determines the type of multilevel interactions. The nearest neighbor ion, dipole–dipole, dipole–quadrupole, and quadrupole–quadrupole interactions are represented severally by = 3, 6, 8, and 10 [26]. To probe into the values of *θ*, we chose *x* = 0.03, 0.06, 0.09, 0.12, 0.15, 0.18, and 0.21 samples corresponding to the emission intensity for the calculation. A linear plot of lg(I/x) versus lg(x) in Ca_1.8_Li_0.6_La_0.6__−x_(PO_4_)_2_:xDy^3+^ is shown in Figure 5b. Fitting the data reveals that the slope is −1.661, resulting in the value of *θ* being 4.983, which is near to 6. The main mechanism of concentration quenching in the sample is hence dipole–dipole energy transfer.

**Figure 6:**
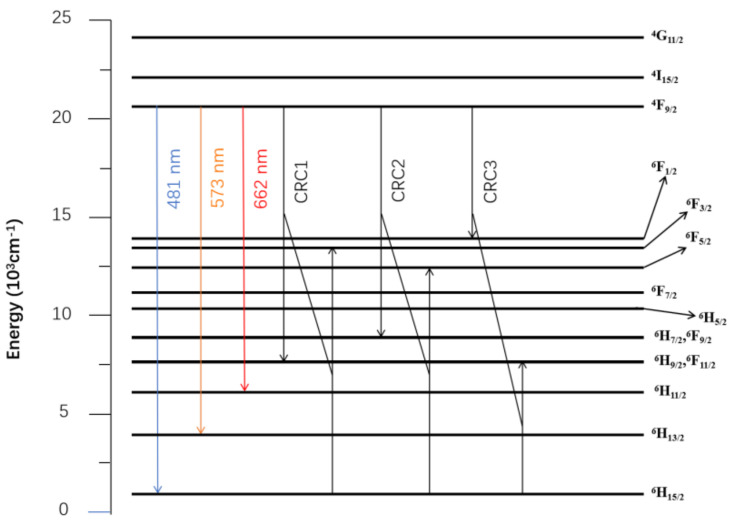
The energy level scheme and the three cross-reaction mechanisms of Dy^3+^.

Asymmetric molecules have intrinsic dipoles due to the uneven spatial distribution of electrons around the positively charged nucleus. The dipole–dipole interaction is calculated from the potential energy of the two dipoles, and the magnitude of the interaction depends on the central distance of the two dipoles and their relative orientation. After the activator concentration becomes large, a strong energy exchange occurs between activators, and this energy exchange depletes the number of luminescent energy levels and weakens the fluorescence intensity, which leads to a concentration quenching. The three cross-relaxation processes of Dy^3+^ (CRC1, CRC2, CRC3) are shown in Figure 6. Dy^3+^ in the ^4^F_9/2_ level can be de-excited to the (^6^H_9/2_,^6^F_11/2_), (^6^H_7/2_,^6^F_9/2_), and ^6^F_1/2_ levels, while the ground state Dy^3+^ receives energy to be excited to the ^6^F_3/2_, ^6^F_5/2_, and (^6^H_9/2_,^6^F_11/2_) levels [3].

### 3.4. Thermal Stability

**Figure 7:**
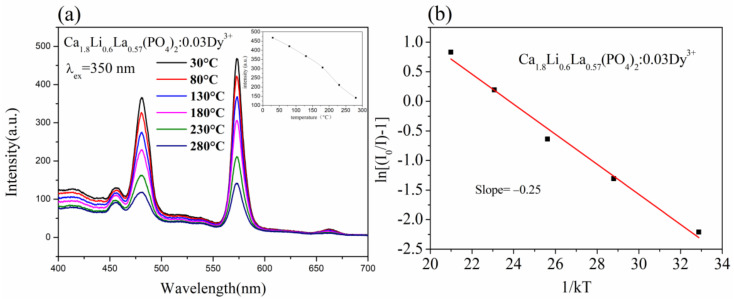
(**a**) The temperature-dependent photoluminescence emission spectra of Ca_1.8_Li_0.6_La_0.57_(PO_4_)_2_:0.03Dy^3+^ phosphor(λex = 350 nm) and (**b**) the relationship between *ln*[(*I*_0_/*I*)−1] and 1/*kT* of Ca_1.8_Li_0.6_La_0.57_(PO_4_)_2_:0.03Dy^3+^ phosphor.

To investigate the samples’ thermal stability, we used variable temperature spectroscopy on the sample with the best luminescence intensity, Ca_1.8_Li_0.6_La_0.57_(PO_4_)_2_: 0.03Dy^3+^. Figure 7a shows the photoluminescence emission spectra (λ_ex_ = 350 nm) at variable temperatures ranging from 30 to 280 °C for Ca_1.8_Li_0.6_La_0.57_(PO_4_)_2_:0.03Dy^3+^. The intensity values of the highest emission peaks at different temperatures are shown in the insets in Figure 7a. The luminescence intensity of the sample steadily drops as the temperature rises. At 130 °C, the luminescence intensity of the sample was about 78.7% of the initial intensity at room temperature, and at 180 °C, it was about 65.5 % of the initial intensity at room temperature.

The activation energy of the sample was derived from the experimental data using the Arrhenius formula to further study the sample’s thermal stability:

(4) $$I=I_{0}/\left[1+A\exp(-\Delta E/kT)\right]$$

where *T* is the temperature, *I*_0_ is the starting luminous intensity, *I* is the luminous intensity at *T*, ∆*E* is the activation energy, *A* is a constant, and k is the Boltzmann constant (8.617 × 10^−5^ eV) [27]. The relationship between ln[(*I*_0_/*I*) − 1] and 1/*kT* is shown in Figure 7b, and the Ca_1.8_Li_0.6_La_0.57_(PO_4_)_2_:0.03Dy^3+^ sample’s activation energy was 0.25 eV. Because a greater ∆*E* raises the nonradiative barrier between the excited and ground states, the higher the value of ∆*E*, the better the phosphor’s thermal stability. The sample has a higher ∆*E* value compared to SrLu(PO_4_)_3_:Dy^3+^ with ∆*E* = 0.214 eV [3], which means that the sample has a better thermal stability. In conclusion, the thermal stability of Ca_1.8_Li_0.6_La_0.57_(PO_4_)_2_:0.03Dy^3+^ phosphor is excellent.

By comparing the results of this article with the other work [2,3,28,29,30], we obtained Table 4. It can be seen that Ca_1.8_Li_0.6_La_0.6__−x_(PO_4_)_2_:xDy^3+^ emits a more standard white light, and it has better thermal stability.

## 4. Conclusions

By replacing Ca^2+^ in whitlockite with Li^+^ and La^3+^, the high-temperature solid state technique was used to generate Ca_1.8_Li_0.6_La_0.6__−x_(PO_4_)_2_:xDy^3+^, a whitlockite luminous material with good luminescence characteristics. First, the X-ray diffraction patterns of the synthesized samples were examined to ensure that they were pure phases. Then, a series of characterizations of the luminescence properties of these samples were carried out. From the experimental data, it is clear that under the excitation of 350 nm UV light, the samples emit white light with two main strong emission peaks at 481 nm in the blue band and 573 nm in the yellow band, which correspond to the electronic transitions at ^4^F_9/2_→^6^H_15/2_ and ^4^F_9/2_→^6^H_13/2_ of Dy^3+^, respectively. Concentration quenching occurred when the Dy^3+^ doping concentration was 0.03. The analysis found that the main source of the samples’ concentration quenching was dipole–dipole energy transfer. At room temperature, the luminescent material emits white light with CIE coordinates of (0.2750, 0.3006). Ca_1.8_Li_0.6_La_0.6__−x_(PO_4_)_2_:xDy^3+^ has good thermal stability, and when the temperature reaches 130°C, the luminescence intensity of the sample was 78.7% of the initial intensity at ambient temperature, and their thermal quenching activation energy was 0.25 eV. As a new type of single-component white phosphor, the synthesis process of Ca_1.8_Li_0.6_La_0.6__−x_(PO_4_)_2_:xDy^3+^ is relatively simple, the synthesis technology requirement is low, and it has good luminescence performance and thermal stability. Therefore, it has good scientific value and social benefits, and it has great economic prospects in the field of phosphors for LED lights.

## Figures and Tables

**Table 1:** Quality of raw materials for Ca_1.8_Li_0.6_La_0.6__−x_(PO_4_)_2_:xDy^3+^.

Sample	x = 0.00	x = 0.01	x = 0.03	x = 0.06	x = 0.09	x = 0.12	x = 0.15	x = 0.18	x = 0.21
Li_2_CO_3_ (g)	0.0443	0.0443	0.0443	0.0443	0.0443	0.0443	0.0443	0.0443	0.0443
La_2_O_3_ (g)	0.1955	0.1922	0.1857	0.1759	0.1662	0.1564	0.1466	0.1368	0.1271
CaCO_3_ (g)	0.3603	0.3603	0.3603	0.3603	0.3603	0.3603	0.3603	0.3603	0.3603
Dy_2_O_3_ (g)	0.0000	0.0037	0.0112	0.0224	0.0336	0.0448	0.0559	0.0671	0.0783
NH_4_H_2_PO_4_ (g)	0.4601	0.4601	0.4601	0.4601	0.4601	0.4601	0.4601	0.4601	0.4601

**Table 2:** Lattice parameters of Ca_1.8_Li_0.6_La_0.6__−x_(PO_4_)_2_:xDy^3+^.

Sample	Standard	x = 0	x = 0.01	x = 0.03	x = 0.06	x = 0.09	x = 0.12	x = 0.15	x = 0.18	x = 0.21
a (Å)	10.429	10.435	10.411	10.407	10.419	10.431	10.423	10.428	10.412	10.402
c (Å)	37.380	37.349	37.433	37.418	37.319	37.354	37.278	37.270	37.232	37.173
Cell volume (Å)	3520.90	3521.97	3513.70	3509.69	3508.22	3519.77	3507.12	3510.05	3495.80	3483.42

**Table 3:** CIE coordinates of Ca_1.8_Li_0.6_La_0.6__−x_(PO_4_)_2_:xDy^3+^ at different temperatures.

Temperature (°C)	X	Y
30 °C	0.2750	0.3006
80 °C	0.2753	0.3005
130 °C	0.2756	0.3004
180 °C	0.2738	0.2974
230 °C	0.2668	0.2885
280 °C	0.2568	0.2754

**Table 4:** Comparison of the results of this article with the other work.

	Ca_1.8_Li_0.6_La_0.6__−x_(PO_4_)_2_:xDy^3+^	Ba_3_Bi(PO_4_)_3_:Dy^3+^, Eu^3+^	SrLu(PO_4_)_3_:Dy^3+^	K_3_ZnB_5_O_10_:Dy^3+^	CaSr_2_(PO_4_)_2_:Dy^3+^, Li^+^	NaLa(PO_3_)_4_:Dy^3+^
Concentration quenching	x = 0.03	x = 0.08	x = 0.08	x = 0.05	x = 0.06	x = 0.06
CIE Coordinate	(0.2750, 0.3006)	(0.3920, 0.3780)	(0.3740, 0.4070)	(0.2560, 0.2580)	(0.3450, 0.3787)	(0.2923, 0.3359)
Activation energy	0.250 eV	0.230 eV	0.214 eV	0.520 eV	Unspecified	Unspecified

## Data Availability

The study does not report any data.
